# Highly Active AuCu-Based Catalysts for Acetylene Hydrochlorination Prepared Using Organic Aqua Regia

**DOI:** 10.3390/ma12081310

**Published:** 2019-04-22

**Authors:** Haihua He, Jia Zhao, Bolin Wang, Yuxue Yue, Gangfeng Sheng, Qingtao Wang, Lu Yu, Zhong-Ting Hu, Xiaonian Li

**Affiliations:** 1 Industrial Catalysis Institute, Laboratory Breeding Base of Green Chemistry-Synthesis Technology, Zhejiang University of Technology, Hangzhou 310014, China; hehaihua2002@jhc.edu.cn (H.H.); wbl501028@outlook.com (B.W.); yueyuxue@outlook.com (Y.Y.); shenggangfeng12@outlook.com (G.S.); qtwang@zjut.edu.cn (Q.W.); yuluuni2014@gmail.com (L.Y.); 2 Pharmaceutical and Material Engineering School, Jin Hua Polytechnic, Jinhua 321007, China; 3 College of Environment, Zhejiang University of Technology, Hangzhou 310014, China; zthu@zjut.edu.cn

**Keywords:** acetylene hydrochlorination, AuCu catalyst, organic aqua regia (OAR)

## Abstract

Development of a sustainable process for designing and synthesising an active and stable catalyst for hydrochlorination of acetylene is challenging, yet crucial, for industrial vinyl chloride monomer (VCM) production. Herein, direct synthesis of bimetallic AuCu catalysts using organic aqua regia (OAR) preparation methods was investigated. In comparison with conventional aqua regia (AR), bimetallic AuCu catalysts synthesised from OAR exhibit enhanced activity and stability. After careful characterisation of the catalyst samples using X-ray diffraction patterns (XRD), Scanning transmission electron microscopy (STEM), X-ray photoelectron spectroscopy (XPS), and Temperature-programmed desorption (TPD), this observation was justified for the following reasons: 1) the existence of sulphur and nitrogen atoms stabilised the cationic Au active sites, and 2) OAR helped to sustain the function of the Cu promotor by stabilising it. Advanced understanding on the importance of promoter stability has unveiled new perspectives for this research area.

## 1. Introduction

Acetylene hydrochlorination (C_2_H_2_ + HCl → CH_2_=CHCl, △H = −124.8 kJ/mol) is an important reaction for producing vinyl chloride monomer (VCM) for chemical processing [1]. However, the most universal catalyst for this reaction, mercury chloride, is highly volatile, toxic and persistent [2,3], and can pose a serious threat to human health and the environment, thus non-mercury analogues are urgently required. To obtain a promising and practical non-mercury alternative, various catalysts include noble metals [4,5,6,7,8,9,10,11,12,13,14,15,16,17,18,19,20], non-noble metals [21,22,23,24] and even non-metal materials [25,26,27,28,29,30,31,32] have been investigated. Pioneering work by Hutchings revealed that gold in the ionic state possesses a unique catalytic activity on the hydrochlorination reaction of acetylene [4,5,6,7,8,9,10]. Thereafter, catalysts with high efficiency in the form of Au/carbon demonstrate that the gold is feasible as a substitute for the poisonous HgCl_2_ catalyst [33,34,35,36,37,38,39,40]. Despite the impressive success achieved, the easy deactivation of Au^3+^ catalyst originated from its high standard electrode potential [41,42] and sintering of the catalyst [12], which largely restrains its application in industry.

The design of the catalyst has so far been the main approach to improve the reaction efficiency of acetylene hydrochlorination. As a potential replacement for Hg-based catalyst, a variety of efforts have been tried to improve the catalytic performance of Au-based catalysts, common strategies include 1. Doping the catalyst supports with heteroatoms such as N [43,44,45,46], S [47], and B [48]; 2. Addition of a second metal like Cs [49,50], Co [51], La [52], Ni [53], Ce [54], In [55] or Cu [56,57,58,59,60,61,62,63]. Bimetallic AuCu catalysts showing satisfying catalytic performance are among the most attractive catalytic systems discovered so far, but like all the other Au-based catalysts, it still suffers from its limited lifetime caused by reduction of cationic Au^3+^ to metallic Au^0^. Therefore, here we aim to gain a deeper understanding on the effect of promoters to facilitate the development of catalytic systems with enhanced performance.

In our previous study, instead of using conventional aqua regia (AR), we have successfully synthesised an active carbon (AC)-supported Au-based catalyst using a new organic aqua regia (OAR) prepared by adding organic compounds to SOCl_2_ [64]. In this work, we demonstrated that bimetallic AuCu catalysts can be prepared via this OAR approach. Compared with the synthetic process of the catalyst using aqua regia (AuCu/AC(AR)), we find that the AuCu/AC(OAR) exhibited a very good catalytic performance. It can be observed from the catalyst characterisation that the OAR preparation method can be helpful to prevent reduction of the active Au species because of the presence of the coordinating abilities of the sulfur. More importantly, mechanistic studies revealed that the OAR can also stabilise Cu species to sustain the promoting effect of Cu. These results are important for the design of active and stable bimetallic Au-based catalysts.

## 2. Materials and Methods 

### 2.1. Catalyst Preparation

Activated carbon (Norit ROX 0.8, Cabot Cor., Boston, MA, USA) was employed as the catalyst support. The AuCu/AC(OAR) catalyst was synthetised by using the wet impregnation method and OAR was used as a solvent. In a typical synthesis of the AuCu_1_/AC(OAR) catalyst, 0.1 mL of HAuCl_4_ (0.06 gAu/mL, Reagent No.1 Factory of Shanghai Chemical Reagent Co., Ltd., Shanghai, China) and 1 mL of CuCl_2_ (0.03 g Cu/mL, Shanghai Titanchem Co., Ltd., Shanghai, China) aqueous solution were injected into 5.4 mL of OAR (1:10 thionyl chloride (SOCl_2_, AR, Shanghai Ling Feng Reagent Co., Ltd., Shanghai, China): N,N-Dimethylformamide (DMF, AR, Shanghai Ling Feng Reagent Co., Ltd., Shanghai, China)) with a magnetic stirrer. The solution was stirred for 2 h and then injected into 2.97 g of activated carbon. The mixture was agitated via stirring and then drying at 110 °C under vacuum for 16 h. AuCu/AC(OAR) catalysts with various Cu loadings (0.2 wt% to 2 wt%) were synthesised using the same synthetic procedures, varying the amount of CuCl_2_ solution added. Au loading was kept constant in all catalysts at 0.2 wt%, and the obtained bimetallic AuCu catalysts were labeled as AuCu_x_/AC(OAR) with the x equivalent to the Cu loading (0.2, 0.6, 1 and 2 wt%). For comparison, the bimetallic AuCu_1_/AC(AR) catalyst was also prepared with the same procedure mentioned above, but used aqua regia (1:3 HNO_3_ (>68%, Shanghai Ling Feng Reagent Co., Ltd., Shanghai, China ): HCl (>37%, Shanghai Ling Feng Reagent Co., Ltd., Shanghai, China)) as a solvent. This was used as a reference catalyst.

### 2.2. Catalyst Characterisation

Morphology and microstructures of the catalysts were characterised using transmission electron microscopy (TEM). A Tecnai G2 F30 S-Twin (Thermo Scientific, Waltham, MA, USA) electron microscope was used for TEM observation. X-ray photoelectron spectroscopy (XPS) analysis was performed with a Kratos AXIS Ultra DLD (Shimadzu Corp., Tokyo, Japan) apparatus, equipped with monochromatised aluminum X-ray source, and passed energy with an electron analyzer of 40 eV. The spectra were corrected for charging using the C 1s binding energy (BE) as the reference at 284.8 eV. Brunauer-Emmertt–Teller (BET) specific surface areas were measured using N_2_ adsorption–desorption at 77 K in a Micromeritics ASAP 2000 (Micromeritics Instruments Corp., Norcross, GA, USA)apparatus. The TPD experiments were conducted in a tubular quartz reactor. A total of 75 mg of each catalyst sample was initially treated with pure C_2_H_2_ or HCl at 180 °C for 30 min after the adsorption, sweeping with pure Ar at gas flow rate of 30 mL/min for 60 min to blow the sample at room temperature (25 °C). Then, a temperature-programmed route was carried out from 25 °C to 550 °C at a heating rate of 10 °C /min. 

### 2.3. Catalytic Test

The catalytic performance of the above-mentioned catalysts were measured in a fixed bed microreactor in which 0.2 g of sample mixed with 0.3 g quartz sand was supported. A reaction gas with a mixture of C_2_H_2_ and HCl was used to give a fed volume ratio V(HCl)/V(C_2_H_2_) of 1.2 and a space velocity of 1480 h^−1^. The effluent gas was detected on-line with a Fuli 9790 GC (Zhejiang Fuli Co., Ltd., Wenling, China). Prior to analysis, the reaction products were first passed through an absorption vessel containing NaOH solution to remove the unreacted hydrogen chloride. 

## 3. Results and Discussion

### 3.1. Optimisations for the Bimetallic Au-Based Catalysts

**Figure 1:**
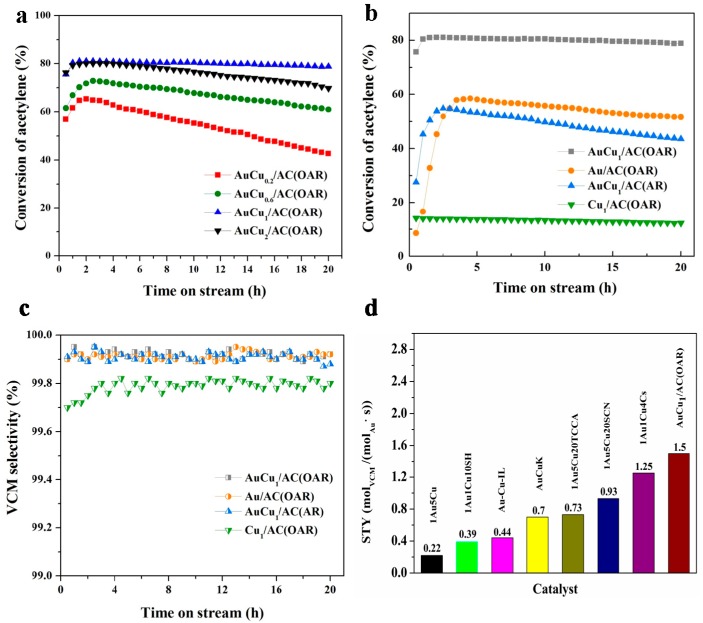
(**a**) The effect of Cu addition on the catalytic performance of AuCu/AC(OAR) catalysts. (**b**) Conversion of C_2_H_2_ and (**c**) selectivity to VCM in hydrochlorination of acetylene over AuCu_1_/AC(OAR), AuCu_1_/AC(AR), Au/AC(OAR) and Cu_1_/AC(OAR). (**d**) The STY values for different bimetallic AuCu catalytic systems: 1Au5Cu [57], 1Au1Cu10SH [66], Au-Cu-IL [40], AuCuK [58], 1Au5Cu20TCCA [62], 1Au5Cu20SCN [34], 1Au1Cu4Cs [56] and AuCu_1_/AC(OAR). Reaction conditions: T = 180 °C, C_2_H_2_ GHSV = 1480 h^−1^, feed volume ratio V(HCl)/V(C_2_H_2_) = 1.2.

Catalytic performance of AuCu/AC(OAR) samples with fixed Au loading but different Cu loading (Cu loading = 0.2, 0.6, 1, 2 wt%) were assessed to investigate the relationship between Cu loading and the activity of catalysts (Figure 1a). Acetylene conversion obtained after reacting for 2 h was increased from 62.3% to 81.2% with the Cu content raised from 0.2 to 1 wt%. Further increase in the Cu loading from 1 to 2 wt% had a negative impact on the stability of the catalyst. Therefore, 1 wt% was determined to be the optimal Cu loading for the AuCu/AC(OAR) catalysts. 

To evaluate the effect of the OAR and Cu promotor on the catalytic performance, acetylene conversions over time for four different catalysts-AuCu_1_/AC(OAR), Au/AC(OAR), AuCu_1_/AC(AR) and Cu/AC(OAR) were recorded for comparison (Figure 1b). AuCu_1_/AC(OAR) had the highest catalytic efficiency and unsurpassed stability, which affirmed that the OAR and Cu promotor had a stabilising effect on the catalysts and could effectively improve the catalytic activity of them. Low acetylene conversion of the Cu/AC(OAR) catalyst demonstrated that Cu^2+^ had negligible catalytic activity towards the hydrochlorination reaction, thus Cu^2+^ was functioning mainly as a promotor. For the catalysts tested, all of them were highly selective to VCM (Figure 1c), and the side products 1,2-dichloroethane and chlorinated oligomers were present in trace amounts only.

In comparison with other literature on catalytic systems with respect to their corresponding reaction conditions, the leading position of our optimised catalytic system AuCu_1_/AC(OAR) was confirmed (Figure 1d). Space-time yield (STY, mol_VCM_/(mol_Au_·s)) results of VCM calculated for AuCu_1_/AC(OAR) was the highest among those reported in the literature on catalytic systems. Note that the STY of AuCu_1_/AC(OAR) was even higher than the state-of-the-art commercial Na_3_Au(S_2_O_3_)/AC catalyst with a Au loading of 0.1 wt% [65], indicating that the practical application of AuCu_1_/AC(OAR) was highly probable. Moreover, the catalytic performance of this work and others are also listed in Table S1, which further indicates the potential application value of the catalyst AuCu_1_/AC(OAR).

### 3.2. Effect of the OAR on the AuCu/AC(OAR) Catalyst

**Figure 2:**
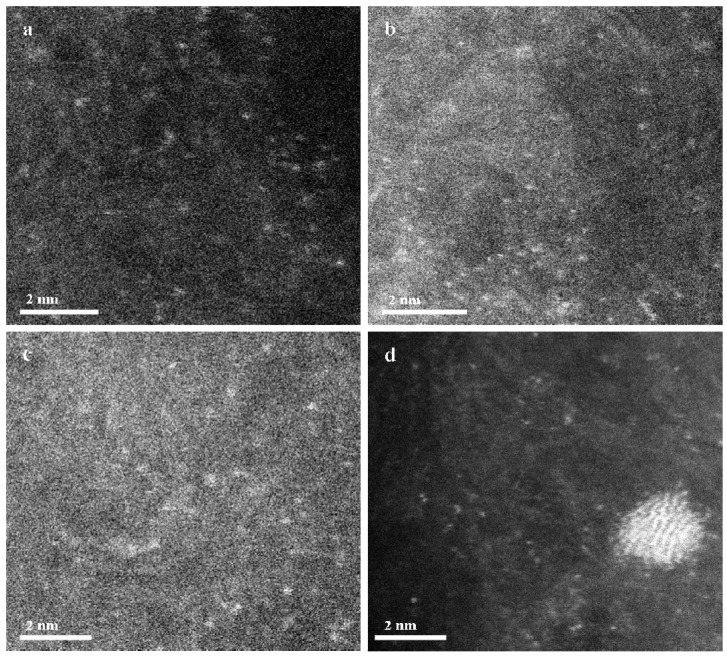
HAADF-STEM images of the fresh and used Au-based catalysts: (**a**) fresh AuCu_1_/AC(OAR), (**b**) fresh AuCu_1_/AC(AR), (**c**) used AuCu_1_/AC(OAR) and (**d**) used AuCu_1_/AC(AR).

Concerning the effect of the preparation method on the morphology of the synthesised catalysts, and the catalyst deactivation during the reaction, high-angle annular dark-field scanning transmission electron microscopy (HAADF-STEM) was performed for both fresh and used bimetallic AuCu catalysts synthesised using OAR and aqua regia, respectively (Figure 2). As shown in Figure 2a,b, the HADDF-STEM images for both fresh AuCu_1_/AC(OAR) and AuCu_1_/AC(AR) catalysts, respectively, display isolated bright dots, which can be attributed to the single Au and/or Cu atoms on the AC support. This result is consistent with Malta [4] and Conte [8] who have proven that the impregnation of HAuCl_4_ with aqua regia at a nominal total metal loading of 1 wt% gave a catalyst with atomically dispersed gold in cationic form over the surface of the support and no metallic Au nanoparticles were observed. As shown in Figure S1, EDX analysis indicated the presence of Au, Cu, S and Cl atoms for AuCu_1_/AC(OAR). Moreover, investigation regarding the stability of catalysts synthesised differently was also conducted. For used AuCu_1_/AC(OAR) (Figure 2c), the HAADF-STEM image indicated white points suggestive of a single-site structure without the significant agglomeration having happened. However, the catalyst synthesised using aqua regia showed lower resistance to the sintering effect during the reaction, and there appeared to be an agglomeration of the Au species to some extent (Figure 2d), with some large particles (diameter > 2 nm) being observed. In summary, OAR preparation was proved to be able to inhibit the catalyst sintering during the reaction with a majority of Au species maintaining single-atom identities. Indeed, operando Extended X-ray absorption fine structure (EXAFS) analysis performed by Malta et al. have demonstrated that the active sites are isolated Au ions [4]. The thermally stable single atom AuCu_1_/AC(OAR) exhibited significantly enhanced stability and performance for the hydrochlorination of acetylene. 

XPS analysis was employed to inspect the chemical composition of the AuCu catalysts (Table 1) for both fresh and used catalysts, and found that concentrations of sulphur and nitrogen residues detected on the surface of the catalysts prepared using OAR were relatively high with respect to the catalysts prepared using conventional aqua regia. Further investigation into the nitrogen spectra (Figure S2) of the AuCu_1_/AC(OAR) catalyst illustrated that two distinct nitrogen states were present, and these two states are represented by two lines on the spectra, namely oxygenated N species and pyrrolic N species [67,68]. Besides, two peaks corresponding to two different sulphur states were also observed on the sulfur spectra (Figure S3): the peak at 166.8 eV indicated the presence of –SO_n_– [69], while the peak at 162.7 eV might have originated from Au-S [70]. The stabilising effect of these sulphur and nitrogen residues on the AuCu_1_/AC(OAR) catalyst was also supported by previous literature reports ascribing the enhanced activity and stability of N and/or S-doped Au/AC catalysts in contrast with their unmodified analogues [43,44,45,46,47,71]. Furthermore, comparison between XPS analysis of the fresh and used catalysts showed that neither sulphur nor nitrogen leaching were happening. 

**Figure 3:**
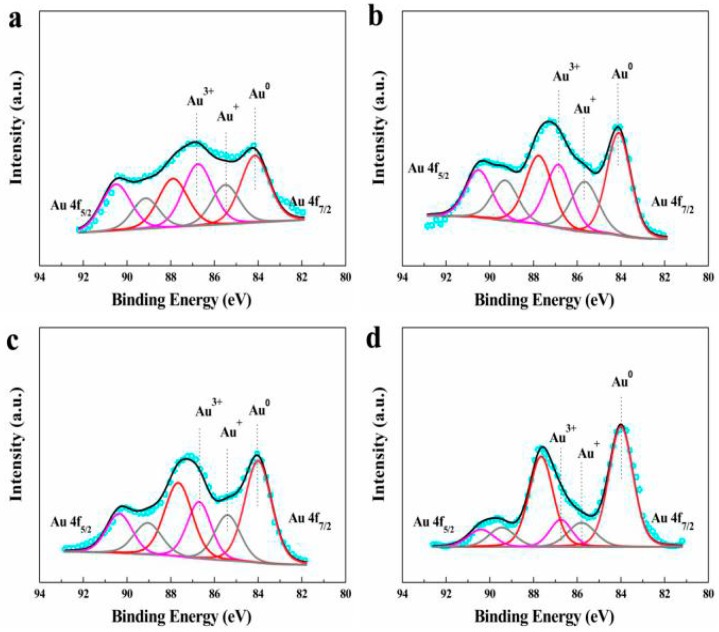
XPS spectrum and simulation for the samples: (**a**) fresh AuCu_1_/AC(OAR), (**b**) used AuCu_1_/AC(OAR), (**c**) fresh AuCu_1_/AC(AR) and (**d**) used AuCu_1_/AC(AR).

XPS was also employed to investigate the change of the valence-state of Au (Figure 3), and active cationic Au^3+^/Au^+^ species with the relative amount in bimetallic AuCu catalysts (Table 2). Three Au states with Au 4f_7/2_ binding energy (BE) of 84.1, 85.4 and 86.7 eV, which were attributed to metallic Au^0^, low-valent Au^+^ and high-valent Au^3+^, respectively, were detected. Relative amount of the cationic Au species in the fresh AuCu_1_/AC(OAR) and AuCu_1_/AC(AR) was 63.4% and 55.2%, respectively; thus, AuCu_1_/AC(OAR) contained more active cationic Au species than AuCu_1_/AC(AR). This observation was owed to the stabilising effect of sulphur atoms on the cationic Au species with a higher valence-state, and it prevented the rapid reduction of those active Au species during catalyst preparation processes and also during the catalytic reaction [65]. As mentioned before, cationic Au species can be reduced to Au^0^ during the reaction leading to catalyst deactivation, for AuCu_1_/AC(AR) catalysts, the percentage composition of the cationic Au species in used was expressively from 55.2% (Table 2, fresh AuCu_1_/AC(AR)) to 31.6% (Table 2, used AuCu_1_/AC(AR)), whereas for AuCu_1_/AC(OAR), the decrease was less dramatic (63.4% to 48.2%, Table 2, fresh and used AuCu_1_/AC(OAR)), which can serve as further evidence to support the fact that OAR synthetic methods can restrain the reduction of cationic Au species, both during the preparation and using processes. 

**Figure 4:**
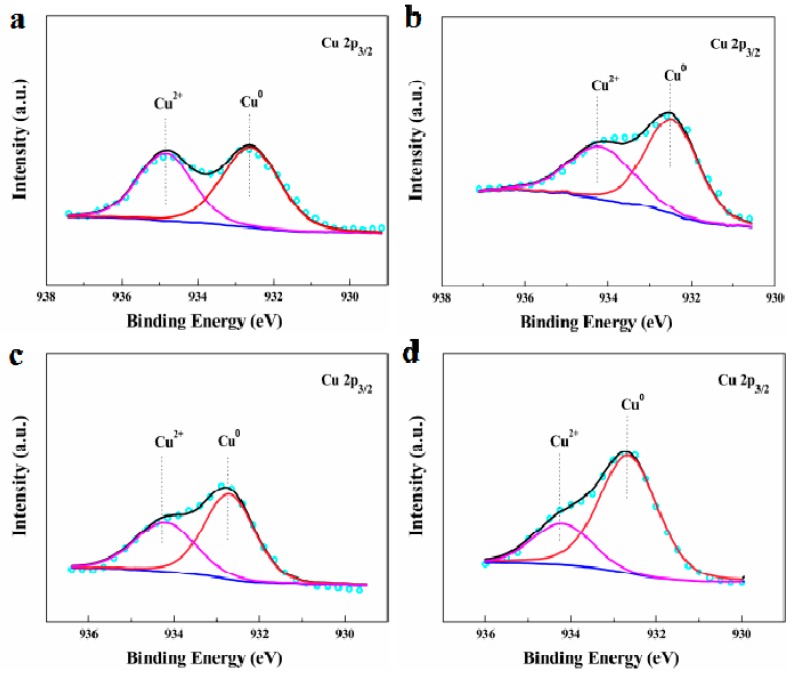
XPS spectra and simulation of the fresh (**a**,**c**) and used (**b**,**d**) AuCu catalysts. (**a**,**b**) AuCu_1_/AC(OAR); (**c**,**d**) AuCu_1_/AC(AR).

After investigation of the Au species, similar examinations were conducted on the other metal content in the catalysts, namely the Cu(II) promotor. Figure 4 presents the XPS spectra of the Cu 2p core levels and the results revealed the presence of Cu^2+^ (BE = 934.1 eV) and Cu^0^ (BE = 931.7 eV) states. It can be clearly seen that a certain amount of Cu^0^ species could also be found from the sample treated using aqua regia or OAR, which can be explained by the adsorption process by AC being accompanied by the reduction of the Cu(II) species. As can be seen, the results obtained for the Cu species were very similar to that for Au, where the percentage composition of Cu^2+^ in AuCu_1_/AC(OAR) (Table 3, fresh and used AuCu_1_/AC(OAR)) was higher than that in AuCu_1_/AC(AR) (Table 3, fresh and used AuCu_1_/AC(AR)), with a lower rate of catalyst deactivation (Figure S4). Hence, as well as for cationic Au species, N and/or S also had a stabilising effect on the Cu species. More importantly, after the reaction, the reduction rate for Au^n+^ (n = 1, 3) and Cu^2+^ were 23.97% and 10.36% for AuCu_1_/AC(OAR), while it was 44.56% and 40.74% for AuCu_1_/AC(AR), respectively (Figure S4). The reduction of cationic Au species to metallic Au was accompanied by the corresponding reduction of Cu^2+^. However, it should be note that the biggest difference between AuCu_1_/AC(OAR) and AuCu_1_/AC(AR) was that AuCu_1_/AC(OAR) had higher nitrogen (≈1.41%) and sulfur (≈1.35%) content, which was consistent with the results in Table 1. Obviously, although the content of Au^n+^ (n = 1, 3) and Cu^2+^ showed a downward trend for both AuCu_1_/AC(OAR) and AuCu_1_/AC(AR) catalysts in the whole reaction process, which was due to the strong reduction of acetylene, the abundant nitrogen and sulfur species on the surface of AuCu_1_/AC(OAR) catalysts still inhibited the forced reduction of Au^n+^ (n = 1, 3) and Cu^2+^ by acetylene. To a certain extent, compared with traditional AuCu_1_/AC(AR) catalysts, the metal active species were stabilised and then improved. In addition, nitrogen and sulfur species not only stabilised the active sites of metals (Au and Cu), but also redisperse the gold species, which is consistent with the results of TEM (Figure 2). It is well known that Cu^0^ species work as electron donors and electrons can be transferred from Cu to Au in AuCu systems. The transfer of electrons to the empty orbital of Au, which may not only enhance the electron-donating ability of Au but also strengthen the adsorption capacity of the HCl. In addition, Cu^2+^ species present in the AuCu catalysts may worked as an oxidant for reduced Au^0^ species to regenerate Au^3+^ with the assistance of the Cl^−^ ligand to stabilise Au^3+^ species [40].

**Figure 5:**
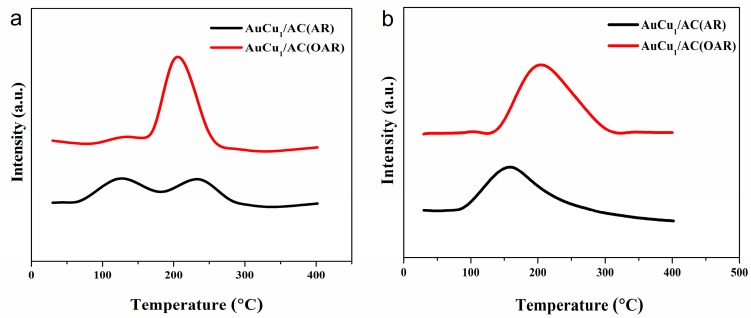
TPD profiles of (**a**) C_2_H_2_ and (**b**) HCl on AuCu_1_/AC(OAR) and AuCu_1_/AC(AR) catalysts.

TPD is an effective characterisation technique for direct comparison of the adsorption capacity of substrates on different catalyst systems [25]. Two desorption peaks are present for acetylene desorption from both AuCu_1_/AC(AR) and AuCu_1_/AC(OAR) catalysts in Figure 5a, where the weaker peaks covering the temperature range of 50–150 °C correspond to the C_2_H_2_ desorption from the carbon support, while the stronger peaks at temperatures higher than 180 °C were attributed to the C_2_H_2_ desorption from cationic Au species. Likewise, from Figure 5b, there was one adsorbed state of HCl on the catalysts at 204 °C for AuCu_1_/AC(OAR). For both the C_2_H_2_- and HCl-TPD spectra, the desorption area of the substrates for AuCu_1_/AC(OAR) was much larger than that of AuCu_1_/AC(AR); nevertheless, the desorption temperature of the substrates for AuCu_1_/AC(OAR) were also different from that for AuCu_1_/AC(AR). The desorption area reveals the amount of substrate adsorbed on the corresponding active sites of the catalysts, where capacity of a catalyst for substrates adsorption is associated with its activity and stability [42]. Furthermore, for the desorption temperature, it is commonly accepted that the weakly bound species usually bear low temperature desorption [25]. These theories in combination with our observations indicated that OAR can undoubtedly aid the generation of catalysts with more active sites and lower the activation energy of acetylene desorption from the catalysts. This can again be explained by the presence of sulphur/nitrogen residues on AuCu_1_/AC(OAR), with the interaction between the residues and Au having a stabilising effect on the active sites, and can also result in different acetylene and hydrogen chloride chemisorption strengths, thus altering the catalytic properties of AuCu_1_/AC(OAR) in contrast to AuCu_1_/AC(AR).

BET analysis was applied to investigate the texture properties of fresh and used catalysts via nitrogen adsorption. Table 4 presents the S_BET_ of the fresh and used AuCu catalysts. It can be seen that after loading the active component, the samples still had a relatively large surface area. However, the used catalysts feature surface areas smaller than those of fresh ones (Table 4). For example, about 16.5% of the surface area of the AuCu_1_/AC(AR) catalyst was lost after the reaction. Loss of specific surface area probably led to the deposition of coke material, which may result in clogged pores and decreased catalyst activity. However, the surface areas of the catalysts lost only 7.6% for the AuCu_1_/AC(OAR). This result indicates that the amount of coke deposition was significantly reduced using an OAR preparation strategy for the bimetallic AuCu/AC catalyst.

## 4. Conclusions

In conclusion, advantageous usage of OAR in comparison with aqua regia during the preparation process of the catalyst was demonstrated to be able to efficiently improve the catalytic performance of the bimetallic AuCu/AC catalysts. The optimised catalyst AuCu_1_/AC(OAR) delivered a stable performance during a 200-h test with a conversion percentage of acetylene reaching more than 98.8% under industrial reaction conditions. After careful characterisation of fresh and used bimetallic catalysts, we concluded that the influence of OAR preparation methods on the activity and stability of catalysts could be ascribed to the stabilising effect of the residual sulphur and nitrogen species. More importantly, mechanistic studies revealed that the OAR residual could also stabilise cationic Cu species to sustain the promoting effect of Cu(II). This work has clearly demonstrated that this route for catalyst design can lead to improved catalytic processes.

## Figures and Tables

**Table 1:** Surface composition of the Au-based catalysts, determined using XPS.

Catalysts	Surface Elemental Composition (wt%)						
	Au4f	C1s	Cl2p	O1s	S2p	N1s	Cu2p
Fresh AuCu_1_/AC(OAR)	0.22	89.68	1.95	4.42	1.35	1.41	0.97
Fresh AuCu_1_/AC(AR)	0.21	91.53	2.28	4.75	0.00	0.31	0.92
Used AuCu_1_/AC(OAR)	0.19	90.07	1.89	4.33	1.25	1.32	0.95
Used AuCu_1_/AC(AR)	0.20	91.33	2.54	4.65	0.00	0.36	0.92

**Table 2:** Quantification and identification of Au species over fresh and used bimetallic AuCu/AC catalysts from XPS data.

Catalysts	Au Species (%)			Binding Energies (eV)		
	Au^3+^	Au^+^	Au^0^	Au^3+^	Au^+^	Au^0^
Fresh AuCu_1_/AC(OAR)	26.8	36.6	36.6	86.7	85.4	84.1
Fresh AuCu_1_/AC(AR)	29.8	25.4	44.8	87.0	85.5	84.0
Used AuCu_1_/AC(OAR)	25.8	22.4	51.8	86.8	85.6	84.1
Used AuCu_1_/AC(AR)	14.5	16.1	69.4	86.8	85.8	84.0

**Table 3:** Quantification and identification of Cu species over fresh and used bimetallic AuCu/AC catalysts from XPS data.

Catalysts	Cu Species (%)		Binding Energies (eV)	
	Cu^2+^	Cu^0^	Cu^2+^	Cu^0^
Fresh AuCu_1_/AC(OAR)	44.4	55.6	934.9	932.8
Fresh AuCu_1_/AC(AR)	40.5	59.5	934.4	932.6
Used AuCu_1_/AC(OAR)	39.8	60.2	934.4	932.8
Used AuCu_1_/AC(AR)	24.0	76.0	934.2	932.6

**Table 4:** Surface areas of AC support and bimetallic AuCu/AC catalyst samples.

Catalysts	S_BET_ (m^2^ g^−1^)		∆S_BET_ (m^2^ g^−1^)
	Fresh	Used	
AC	1162.1	/	/
AuCu_1_/AC(AR)	1005.3	839.1	166.2
AuCu_1_/AC(OAR)	1067.6	986.6	81.0

## Supplementary Materials

The following are available online at https://www.mdpi.com/1996-1944/12/8/1310/s1. Figure S1: EDX spectra of fresh AuCu_1_/AC(OAR) catalyst. Figure S2: High-resolution N 1s spectra of the (**a**) fresh and (**b**) used AuCu_1_/AC(OAR) catalyst. Figure S3: High-resolution S 2p spectra of the (**a**) fresh and (**b**) used AuCu_1_/AC(OAR) catalysts. Figure S4: The reduction rate for cationic Au and Cu species: (**a**) AuCu_1_/AC(OAR) and (**b**) AuCu_1_/AC(AR) catalysts. Table S1: Catalytic performance of different AuCu-based catalysts.
