# Supplementary material for: Highly Active AuCu-Based Catalysts for Acetylene Hydrochlorination Prepared Using Organic Aqua Regia
Source: Materials (Basel). 2019 Apr 22;12(8):1310. doi: 10.3390/ma12081310 (PMC6515409; doi:10.3390/ma12081310)
Supplement: Supplementary file 1 [file materials-12-01310-s001.pdf]

Supporting Online Material for

Highly Active Au-Cu-based Catalysts for Acetylene Hydrochlorination Prepared by Organic Aqua Regia

Haihua He [1,3], Jia Zhao [1,*], Bolin Wang [1], Yuxue Yue [1], Gangfeng Sheng [1], Qingtao Wang [1], Lu Yu [1], Zhong-Ting Hu [2] and Xiaonian Li [1,*]

[1] Industrial Catalysis Institute, Laboratory Breeding Base of Green Chemistry-Synthesis Technology, Zhejiang University of Technology, Hangzhou 310014, China.
[2] College of Environment, Zhejiang University of Technology, Hangzhou 310014, China.
[3] Pharmaceutical and Material Engineering School, Jin Hua Polytechnic, Jinhua 321007, China.
* Correspondence: jiazhao@zjut.edu.cn (Jia zhao); xnli@zjut.edu.cn (Xiaonian Li); Tel.: +86 571 88320002.

Table 1. Catalytic performance of different Au-Cu-based catalysts.

**Catalysts**	**Temperature °C**	**GHSV h^{-1}**	**Maximum Conversion %**	**Deactivation Rate[a] h^{-1}**	**Ref**
Au–Cu/C	170	1200	72	0.92	[1]
Au–Cu/AC	180	1200	89	6.75	[2]
Au–Co(III)–Cu(II)/AC	150	360	99	0.08	[3]
Au/TCCA/AC	180	600	90	0.83	[4]
0.1Au1.0Cu/AC	150	120	99	24.6	[5]
Au-Cu-SH/AC	180	1200	75	1.66	[6]
AuCuCs/AC	180	740	89	0.76	[7]
Au-Cu-IL/AC	180	740	75	−0.25	[8]
$AuCu_1$/AC(OAR)	180	1480	80	0.04	This work

[a] Deactivation rate (h^{-1}) was calculated from ($X_{Maximum}$ % − X_{Stop}%) /(T_{Stop} − $T_{Maximum}$). X represents for the acetylene conversion.

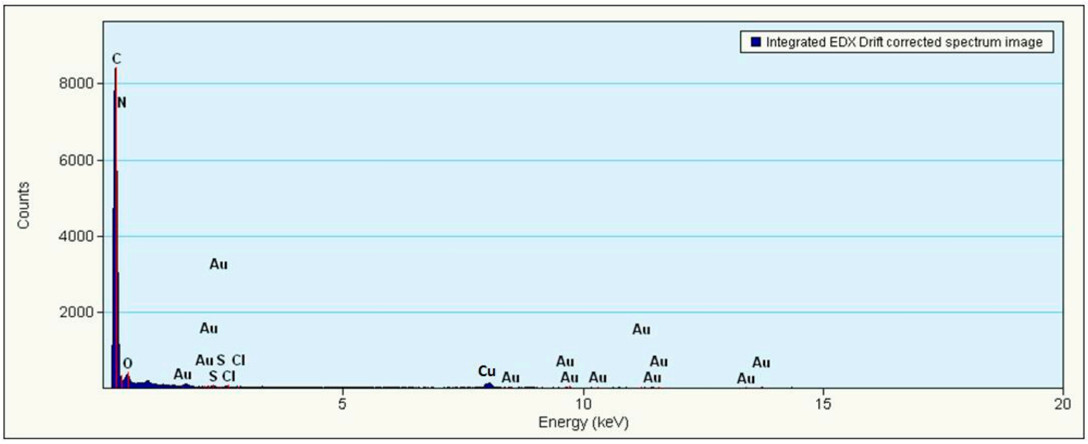

Figure 1. EDX spectra of fresh $AuCu_1/AC(OAR)$ catalyst.

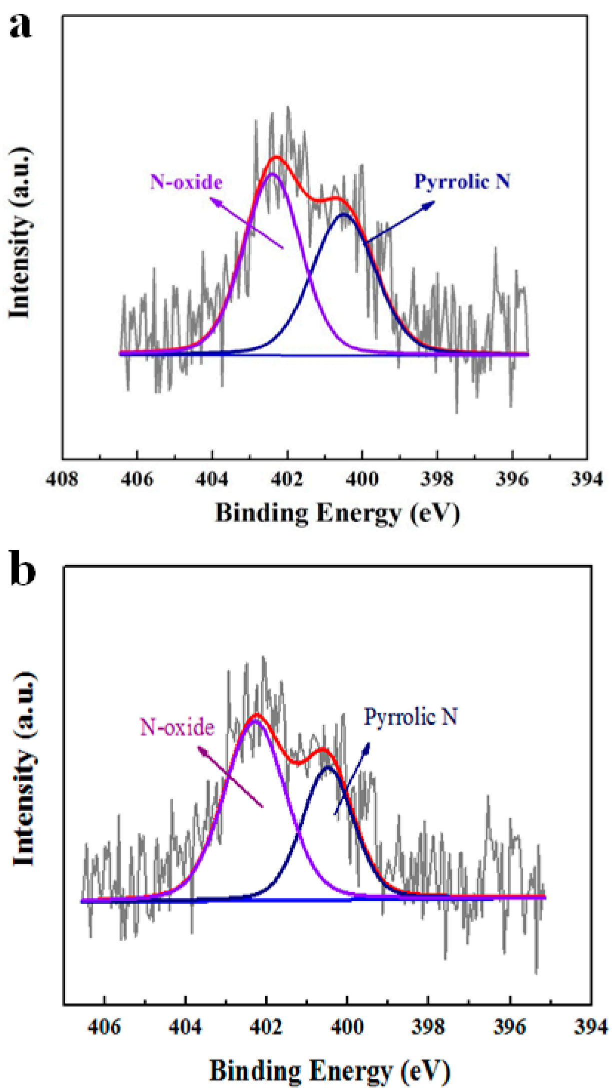

Figure 2. High-resolution N 1s spectra of the (**a**) fresh and (**b**) used $AuCu_1/AC(OAR)$ catalysts.

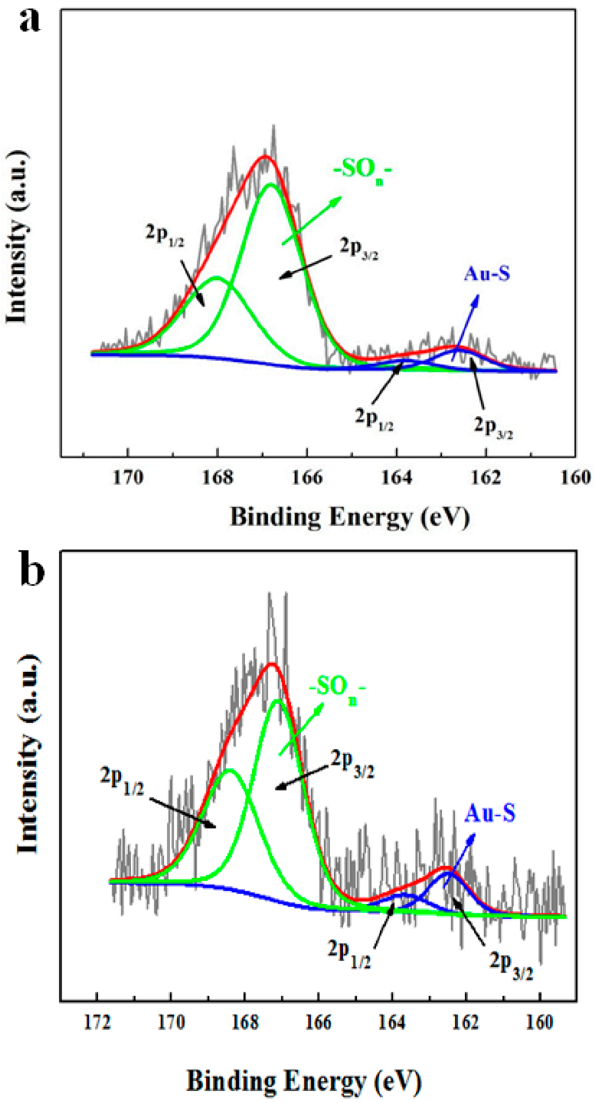

Figure 3. High-resolution S 2p spectra of the (**a**) fresh and (**b**) used $AuCu_1/AC(OAR)$ catalysts.

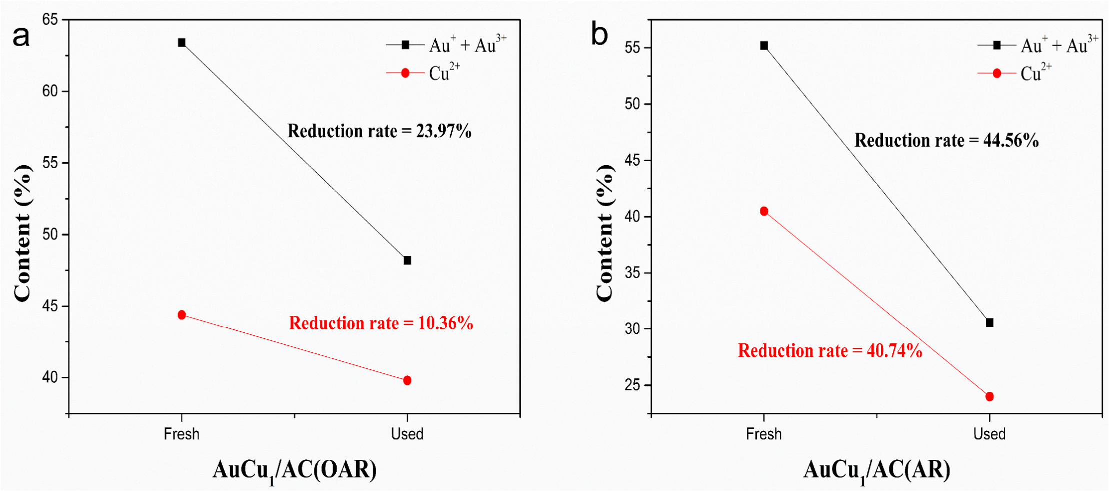

Figure 4. The reduction rate for cationic Au and Cu species: (**a**) AuCu1/AC(OAR) and (**b**) AuCu1/AC(AR) catalysts.

References

1. Wang, S.; Shen, B.; Song, Q. Kinetics of Acetylene Hydrochlorination over Bimetallic Au–Cu/C Catalyst. *Catal. Lett*. **2010**, *134*, 102–109.
2. Zhou, K.; Jia, J.; Li, C.; Xu, H.; Zhou, J.; Luo, G.; Wei, F. A low content Au-based catalyst for hydrochlorination of C_2H_2 and its industrial scale-up for future PVC processes. *Green Chem*. **2015**, *17*, 356–364.
3. Zhang, H.; Dai, B.; Li, W.; Wang, X.; Zhang, J.; Zhu, M.; Gu, J. Non-mercury catalytic acetylene hydrochlorination over spherical activated-carbon-supported Au–Co(III)–Cu(II) catalysts. *J. Catal*. **2014**, *316*, 141–148.
4. Xu, H.; Zhou, K.; Si, J.; Li, C.; Luo, G. A ligand coordination approach for high reaction stability of an Au–Cu bimetallic carbon-based catalyst in the acetylene hydrochlorination process. *Catal. Sci. Technol*. **2016**, *6*, 1357–1366.
5. Zhao, J.; Zeng, J.; Cheng, X.; Wang, L.; Yang, H.; Shen, B. An Au–Cu bimetal catalyst for acetylene hydrochlorination with renewable γ-Al_2O_3 as the support. *RSC Adv*. **2015**, *5*, 16727–16734.
6. Hong, G.; Tian, X.; Jiang, B.; Liao, Z.; Wang, J.; Yang, Y.; Zheng, J. Improvement of performance of a Au–Cu/AC catalyst using thiol for acetylene hydrochlorination reaction. *RSC Adv*. **2016**, *6*, 3806–3814.
7. Zhao, J.; Gu, S.; Xu, X.; Zhang, T.; Di, X.; Pan, Z.; Li, X. Promotional effect of copper(ii) on an activated carbon supported low content bimetallic gold–cesium(i) catalyst in acetylene hydrochlorination. *RSC Adv*. **2015**, *5*, 101427–101436.
8. Zhao, J.; Yu, Y.; Xu, X.; Di, S.; Wang, B.; Xu, H.; Ni, J.; Guo, L.; Pan, Z.; Li, X. Stabilizing Au(III) in supported-ionic-liquid-phase (SILP) catalyst using $CuCl_2$ via a redox mechanism. *Appl. Catal. B Environ*. **2017**, *206*, 175–183.
